# Prerequisites to improve surgical cytoreduction in FIGO stage III/IV epithelial ovarian cancer and subsequent clinical ramifications

**DOI:** 10.1186/s13048-023-01303-1

**Published:** 2023-11-11

**Authors:** Diederick de Jong, Amudha Thangavelu, Timothy Broadhead, Inga Chen, Dermot Burke, Richard Hutson, Racheal Johnson, Angelika Kaufmann, Peter Lodge, David Nugent, Aaron Quyn, Georgios Theophilou, Alexandros Laios

**Affiliations:** 1 Department of Gynaecological Oncology, ESGO Centre of Excellence in advanced ovarian cancer surgery, St. James’s University Hospital, LTHT, Beckett Street, Leeds, LS9 7TF UK; 2 Department of Surgery, Hepatobilliary Surgery and Liver Transplant Service, St. James’s University Hospital LTHT, Leeds, UK; 3 Department of Surgery, Colorectal Surgery Service, St. James’s University Hospital LTHT, Leeds, UK

## Abstract

**Background:**

No residual disease (CC 0) following cytoreductive surgery is pivotal for the prognosis of women with advanced stage epithelial ovarian cancer (EOC). Improving CC 0 resection rates without increasing morbidity and no delay in subsequent chemotherapy favors a better outcome in these women. Prerequisites to facilitate this surgical paradigm shift and subsequent ramifications need to be addressed. This quality improvement study assessed 559 women with advanced EOC who had cytoreductive surgery between January 2014 and December 2019 in our tertiary referral centre. Following implementation of the Enhanced Recovery After Surgery (ERAS) pathway and prehabilitation protocols, the surgical management paradigm in advanced EOC patients shifted towards maximal surgical effort cytoreduction in 2016. Surgical outcome parameters before, during, and after this paradigm shift were compared. The primary outcome measure was residual disease (RD). The secondary outcome parameters were postoperative morbidity, operative time (OT), length of stay (LOS) and progression-free-survival (PFS).

**Results:**

R0 resection rate in patients with advanced EOC increased from 57.3% to 74.4% after the paradigm shift in surgical management whilst peri-operative morbidity and delays in adjuvant chemotherapy were unchanged. The mean OT increased from 133 + 55 min to 197 + 85 min, and postoperative high dependency/intensive care unit (HDU/ICU) admissions increased from 8.1% to 33.1%. The subsequent mean LOS increased from 7.0 + 2.6 to 8.4 + 4.9 days. The median PFS was 33 months. There was no difference for PFS in the three time frames but a trend towards improvement was observed.

**Conclusions:**

Improved CC 0 surgical cytoreduction rates without compromising morbidity in advanced EOC is achievable owing to the right conditions. Maximal effort cytoreductive surgery should solely be carried out in high output tertiary referral centres due to the associated substantial prerequisites and ramifications.

**Supplementary Information:**

The online version contains supplementary material available at 10.1186/s13048-023-01303-1.

## Background

Most women with primary epithelial ovarian, fallopian tube, or peritoneal cancer (EOC) present in the advanced stages of disease [1]. Surgical cytoreduction combined with chemotherapy has been the cornerstone in the treatment of the advanced stages of EOC for more than half a century [2]. It has been demonstrated that prognosis and survival in the advanced stages of EOC is inversely related to residual disease (RD) following cytoreductive surgery [3, 4]. The traditional approach, primary debulking surgery (PDS) to < 1 cm followed by adjuvant chemotherapy, was regarded as the standard of care for decades [5]. Patient prognosis could be improved when surgeons were focusing on improving optimal cytoreduction [6]. More recently, even longer overall survival rates in advanced EOC could be established when no macroscopic residual disease (CC 0) was achieved [7]. Unfortunately, CC 0 resections are not always realized.

Interval debulking surgery (IDS) following neoadjuvant chemotherapy (NACT) was introduced where optimal cytoreduction was felt to be unachievable and/or results in significant morbidity [8]. Evidence from a previous meta-analysis suggests that NACT regimen was associated with inferior survival outcomes [9]. In contrast, the meta-analysis of two prospectively randomized landmark trials showed no difference in survival outcomes between PDS and IDS in patients with advanced EOC [10]. Nevertheless, irrespective of the ongoing debate on the timing of cytoreductive surgery, surgeons who perform cytoreductive surgery for advanced EOC focus on complete cytoreduction as a primary outcome measure in both the PDS and IDS setting.

Maximal effort cytoreductive surgery, aiming at CC 0 resections, in patients with advanced EOC, frequently involves upper abdominal surgery [11, 12]. However, the adoption of this surgical concept for advanced EOC amongst UK Gynecologic Oncologists has been reportedly low [13]. The European Society of Gynaecologic Oncology (ESGO) standardized the quality of (maximal effort) cytoreductive surgery in advanced EOC by formulating 10 quality indicators (QIs) that could impact survival outcomes (Suppl. Table 1) [14].

Historically, survival outcomes for advanced ovarian cancer in our center have been well above the UK average. We previously reported median PFS and OS of 19 months (95% CI 16.4–21.6) and 38 months (95% CI 34.4–41.6), respectively in a previous sub-cohort of advanced EOC patients [15].

Between 2016 and 2017, to improve CC 0 resections and to facilitate more complex multi-visceral surgery, many changes in the surgical management of our patients were introduced. These included: (1) Appointing three consultant colleagues with specific training in (ultra-) radical surgery for EOC; (2) Intensified collaboration with other surgical specialties; (3) Implementation of the optimization of patients’ physical performance program (prehabilitation); (4) Expanding our enhanced recovery (ERAS) program and appointing specialized ERAS nurses; 6) Intensified goal focused peri-operative fluid management; 7) Interdisciplinary pre-operative allocation of available high dependency unit (HDU) and intensive care unit (ICU) postoperative beds; 8) Introduction of the Clavien-Dindo classification for reporting peri-operative complications [16].

This study was designed to assess the impact of surgical management changes on cytoreduction rates in advanced EOC and consequential morbidity and mortality of the surgical procedure. We aimed to compare surgical outcome parameters before during, and after changes in our surgical management were introduced. Subsequently, the conditions for and ramifications of our more aggressive surgical approach in advanced EOC were analyzed. We also evaluated the association between the paradigm shift in advanced EOC surgery and progression-free survival PFS).

## Methods

All patients with FIGO stage III and IV EOC undergoing cytoreductive surgery in our tertiary referral center between 1st January 2014 and 31st December 2019, in either the upfront setting (PDS) or after having received neoadjuvant chemotherapy (IDS) were included in the study. All patients had surgical cytoreduction by a certified and accredited Gynecologic Oncologist. Staging was defined by the 2014 International Federation of Gynaecology and Obstetrics (FIGO) staging system [17]. Excluded were patients with a synchronous primary malignancy and those with recurrent ovarian malignancy. Women with an incidental finding of advanced EOC who had their procedure performed by general surgeons in the emergency setting for bowel obstruction were also excluded.

The surgical outcomes and implications with regards to post-operative recovery in the three-time intervals (2014–2015, 2016–2017, and 2018–2019), before (baseline), during (transition), and after (evaluation) the introduction of our paradigm shift in the surgical management were compared; these time intervals being named baseline, transition, and evaluation years, respectively. Prospectively collected data of these cohorts were retrieved from the hospital wide database Patient Pathway Manager (PPM) [18]. This study was approved by the institutional review board (ID 282396) and performed according to the standards outlined in the Declaration of Helsinki.

In this analysis, the age was defined as age at the time of diagnosis. The Eastern Cooperative Oncology Group (ECOG) Performance Status (PS) [19] and the serum CA125 levels were determined at diagnosis prior to PDS or first course of NACT. All patients had pre-treatment physical examination, serum CA 125 measurement, CT imaging of chest, abdomen and pelvis, and histological diagnosis by either image-guided or surgical biopsy. Results of pre-treatment workup were discussed in our multi-disciplinary team (MDT) followed by a recommendation for upfront surgical cytoreduction or NACT and subsequent IDS. These recommendations were based on PS and dissemination of disease. The full criteria for the timing of cytoreduction have been provided in our recent paper [4].

Following implementation of the ERAS pathway [20] in late 2015, the paradigm shift towards more complex multi-visceral surgery was initiated in the years 2016 and 2017. Prior to the procedure, from 2016 onwards: (1) cardiopulmonary exercise testing (CPET) [21] was requested for patients with PS ≥ 2; (2) All patients had their fitness optimized prior to surgery following implementation of prehabilitation program whenever possible; (3) Patients received lifestyle advice, as well as dietary and medical to support fitness; (4) Planned postoperative HDU/ICU bookings were made based on a previous scoring; [22] (5) Involvement of other surgical specialties was requested when the anticipated required procedure to achieve a complete surgical cytoreduction was not in the skillset of the Gynecologic Oncologist.

The ‘standard’ surgical cytoreduction (total hysterectomy, bilateral salpingo-oophorectomy and omentectomy) could be extended to stripping or resection of diaphragm and peritoneum, stripping of the mesentery, wedge resection of the liver, (partial) gastrectomy, cholecystectomy, splenectomy, pancreas tail resection, adrenalectomy, small and/or large bowel resection with or without stoma formation, appendicectomy, and lymph node dissection in an effort to achieve a complete surgical cytoreduction. On rare occasions, laterally extended endopelvic resection (LEER) [23] or composite exenteration [24] was required to achieve the desired surgical result.

The primary outcome parameter was RD after cytoreductive surgery. Secondary outcome parameters were postoperative morbidity and mortality, number of patients who had PDS in comparison to IDS, intra-operative assessments of dissemination of disease, complexity of the performed surgery, duration of the surgical procedure, intra-operative blood loss, utilization of other surgical specialties, utilization of the HDU or ICU unit, length of hospital stay, delays in starting adjuvant chemotherapy, postoperative morbidity, and mortality, and ESGO QI score [14]. The guidelines for the peri-operative management of advanced EOC patients undergoing debulking surgery were strictly followed [25]. Protocol specifications and management of post-operative complications reflecting the continuous effort to improve the oncologic care of these patients have been described elsewhere [26]. Key components of prehabilitation programs included screening/assessment tools (e.g., frailty, physical activity, mental health, diet) and target interventions (e.g., exercise, psychological support, nutrition). Several types of interventions including their duration are currently under evaluation whilst novel monitoring and evaluation tools are being developed (data not shown).

Residual Disease was categorized according to the size of remaining tumor nodules at the end of the surgical procedure. Complete cytoreduction of tumor was defined as nil RD (CC 0) or 0 mm < RD < 2.5 mm (CC 1), incomplete cytoreduction as 2.5 mm ≤ RD < 2.5 cm (CC 2) or RD ≥ 2.5 cm [27]. This is the preferred way of reporting the RD. Because we work closely with other surgical specialties, we elected to use the well-known Sugarbaker criteria. The CC 1 was measured as RD at the size of a mustard seed or smaller (mostly on the bowel mesentery or serosa). Although all these tiny residuals have been treated with plasmajet, persistent RD albeit very small could not be excluded. Hence, they were categorized as CC 1.

Complexity of the procedure was scored according to the surgical complexity score (SCS) [28]. Peri- and postoperative morbidity and mortality were classified according to the Clavien-Dindo classification [16]. Intraoperative visualization of disease dissemination was scored by the peritoneal cancer index (PCI) [29] and the GOG criteria; the minimal disease (MD) group had tumor limited to the pelvis and retroperitoneal (nodal) metastasis. The abdominal peritoneal disease (APD) group had disease limited to the pelvis and abdomen but excluding the liver, spleen, gallbladder, pancreas, or diaphragm, with or without retroperitoneal spread. The upper abdominal disease (UAD) group had disease affecting the pelvis with or without lower abdominal and retroperitoneal disease, plus involvement of at least one of the following: liver, spleen, gallbladder, pancreas, or diaphragm [30].

Patient characteristics according to group were presented as mean +/- SD, median with range, or absolute numbers with percentages. Differences between the baseline, transition, and evaluation years were analyzed by Chi Square, ANOVA, and Kruskal-Wallis tests, depending on the data distribution. In the absence of a comparator against which the results could be adjusted, we did not consider performing segmented regressions using interrupted time-series. Hence, any changes in the results should be multi-factorial and not essentially attributed to the intervention themselves. Survival data were summarized using the Kaplan–Meier method, and the log-rank test was employed to test significance amongst patient groups for the outcome of PFS. Progression-free survival was defined as the time (months) from the date of diagnosis to the date of progression or recurrence. The patients were followed up until April 2022. Because of the varying time frames, the three groups were subjected to “normalization” for accurate comparison. All tests were two sided and *P* < 0.05 was considered statistically significant for all tests. The software packages Prism 8 (GraphPad Software, San Diego, CA., USA) and SPSS 26 (IBM SPSS Statistics, Armonk, NY., USA) were employed for data analysis.

## Results

**Fig. 1:**
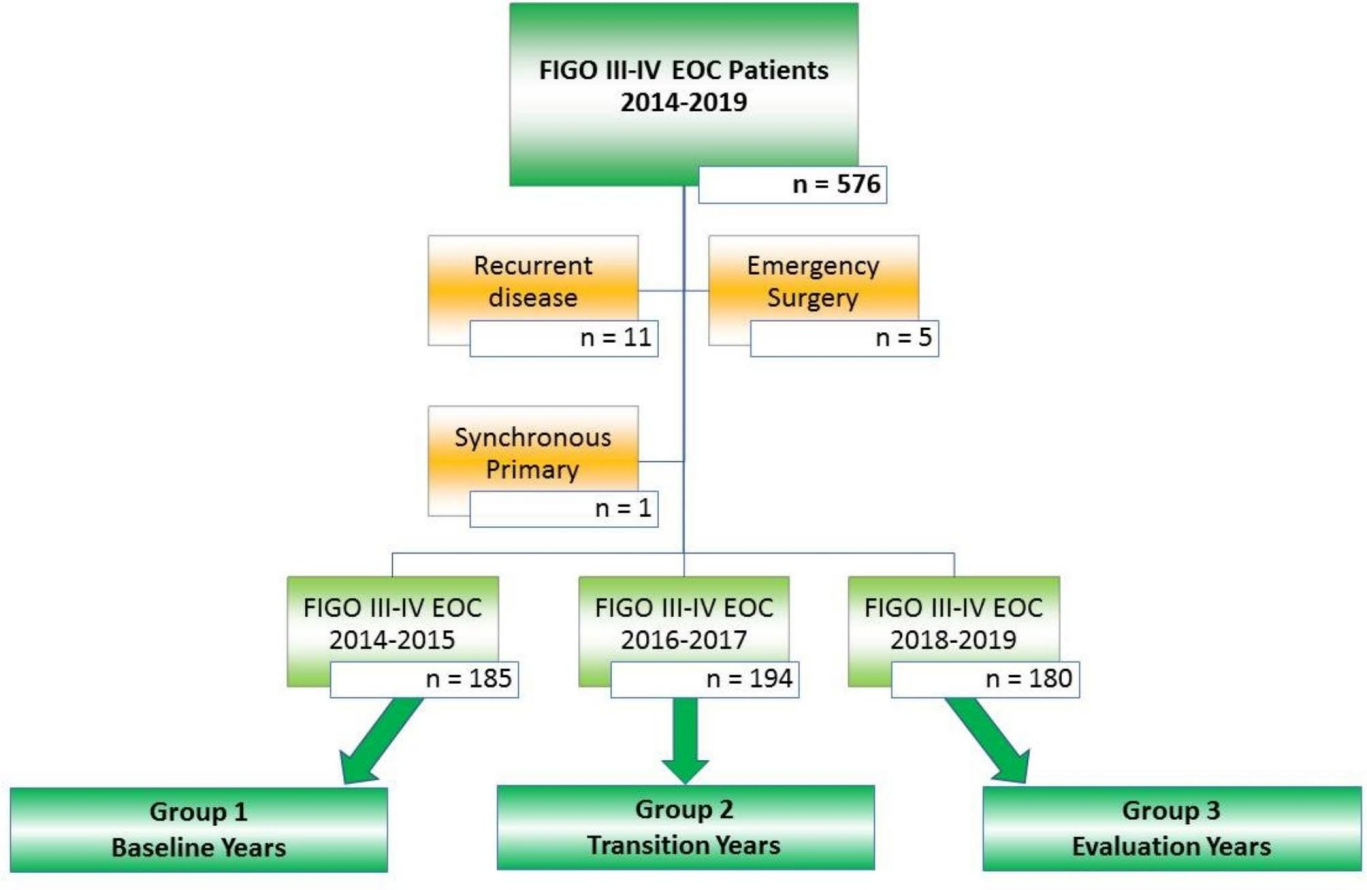
Inclusion and exclusion criteria for all patients with an advanced stage EOC who had cytoreductive surgery between January 2014 and December 2019. Exclusion criteria were applied aiming at a study population of patients who had genuine attempt of cytoreductive surgery of advanced EOC

Between the 1st of January 2014 and the 31st of December 2019, a cohort of 576 consecutive patients with FIGO stage III-IV EOC had surgical resection of tumor bulk either in the upfront or neo-adjuvant setting. We excluded those patients who had surgery for recurrent disease (n = 11), those who had emergency surgery for malignant bowel obstruction by the general surgeons (n = 5), and one patient with a synchronous primary tumor (n = 1). A total of 185 and 194 women had surgical cytoreduction for advanced EOC before and during the introduction and implementation of surgical paradigms aiming at improved CC 0 resections, respectively. One-hundred-eighty women had surgical cytoreduction for advanced EOC following our paradigm shift in the surgical management of ovarian cancer. Details of inclusion and exclusion of patients for this study population are shown in Fig. 1.

The mean age of the patients in the entire cohort was 63.5 + 11.2 years. The mean age was comparable among the groups of patients, 62.7 ± 10.1, 64.1 ± 11.3, and 63.6 ± 12.2 years for patients having surgery during the baseline, transition, and evaluation years, respectively (*P* = 0.296). A PS of 3/4 was observed in 1.7% of the patients undergoing surgical cytoreduction during the evaluation years versus 4.3 and 4.1% during the baseline and transition years, respectively (*P* = 0.033). Other patients’ characteristics were comparable in the different time intervals. Details of baseline characteristics are displayed in Table 1.

**Table 1:** Baseline characteristics of 559 advanced stage (FIGO III-IV) epithelial ovarian cancer (EOC) patients having cytoreductive surgery between the years 2014–2019. 185 before the surgical paradigm shift (baseline years), 194 during the changes (transition years), and 180 patients after the transition (evaluation years). Numbers are shown either as absolute numbers with percentage unless otherwise indicated

Patients	Baseline Years 2014–2015	Transition Years 2016–2017	Evaluation Years 2018–2019	*P*-value
	n = 185	n = 194	n = 180	
**Age (yrs)** Mean ± SD	58.0 ± 7.79	66.3 ± 8.79	64.4 ± 9.79	**0.465**
**Performance**				**0.033**
PS 0	82 (44.3%)	80 (41.2%)	103 (57.2%)	
PS 1	76 (41.1%)	76 (39.2%)	56 (31.1%)	
PS 2	19 (10.3%)	30 (15.5%)	18 (10.0%)	
PS 3/4	8 (4.3%)	8 (4.1%)	3 (1.7%)	
**CA125 (U/mL)** Median (Range)	625 (20-18990)	604 (13-28600)	427 (13-17900)	**0.306**
**Histology**				**0.465**
Serous	161 (87.1%)	170 (87.6%)	151 (83.8%)	
Mucinous	3 (1.6%)	6 (3.1%)	5 (2.8%)	
Clear Cell	9 (4.9%)	4 (2.1%)	4 (2.2%)	
Endometrioid	3 (1.6%)	7 (3.6%)	5 (2.8%)	
Undifferentiated	3 (1.6%)	3 (1.6%)	3 (1.7%)	
Carcinosarcoma	5 (2.7%)	2 (1.0%)	9 (5.0%)	
Other	1 (0.5%)	2 (1.0%)	3 (1.7%)	
**Tumor Grade**				**0.875**
1 (Well Differentiated)	17 (9.2%)	22 (11.3%)	17 (9.4%)	
2 (Moderately Differentiated)	3 (1.6%)	5 (2.6%)	3 (1.7%)	
3 (Poorly Differentiated)	165 (89.2%)	167 (86.1%)	160 (88.9%)	
**FIGO Stage**				**0.478**
III A-B	22 (11.9%)	27 (13.9%)	31 (17.2%)	
III C	115 (62.1%)	114 58.8%)	96 (53.3%)	
IV A-B	48 (26.0%)	53 (27.3%)	53 (29.5%)	
**Ethnicity**				**0.271**
White-British	151 (81.6%)	174 (89.7%)	157 (87.2%)	
White - Other	10 (5.4%)	4 (2.1%)	6 (3.3%)	
South Asian	1 (0.5%)	0 (0.0%)	0 (0.0%)	
South-East Asian	6 (3.3%)	5 (2.6%)	4 (2.2%)	
East Asian	0 (0.0%)	0 (0.0%)	1 (0.6%)	
Black-Caribbean	2 (1.1%)	3 (1.5%)	0 (0.0%)	
Black-African	1 (0.5%)	0 (0.0%)	1 (0.6%)	
Hispanic	2 (1.1%)	1 (0.5%)	0 (0.0%)	
Middle Eastern	0 (0.0%)	0 (0.0%)	2 (1.1%)	
Mixed	7 (3.8%)	2 (1.0%)	2 (1.1%)	
Undisclosed*	5 (2.7%)	5 (2.6%)	7 (3.9%)	

CC 0 resections were achieved in 75.0% of the cases having surgery during the evaluation years versus 56.2% and 65.5% during the baseline and transition years, respectively (*P* = 0.0041). During the baseline years, 11.4% of the cases required the assistance of a gastro-intestinal and/or hepato-biliary surgeon or Gynecologic Oncologist colleague compared to 52.6% and 41.1% in the transition and evaluation years, respectively (*P* < 0.0001). The SCS increased from 3.0 ± 1.3 for the baseline years to 3.9 ± 2.1 and 4.5 ± 2.5 for the transition and evaluation years, respectively (*P* < 0.00001). The average blood loss resulting from the procedure remained unchanged throughout the study period.

**Fig. 2:**
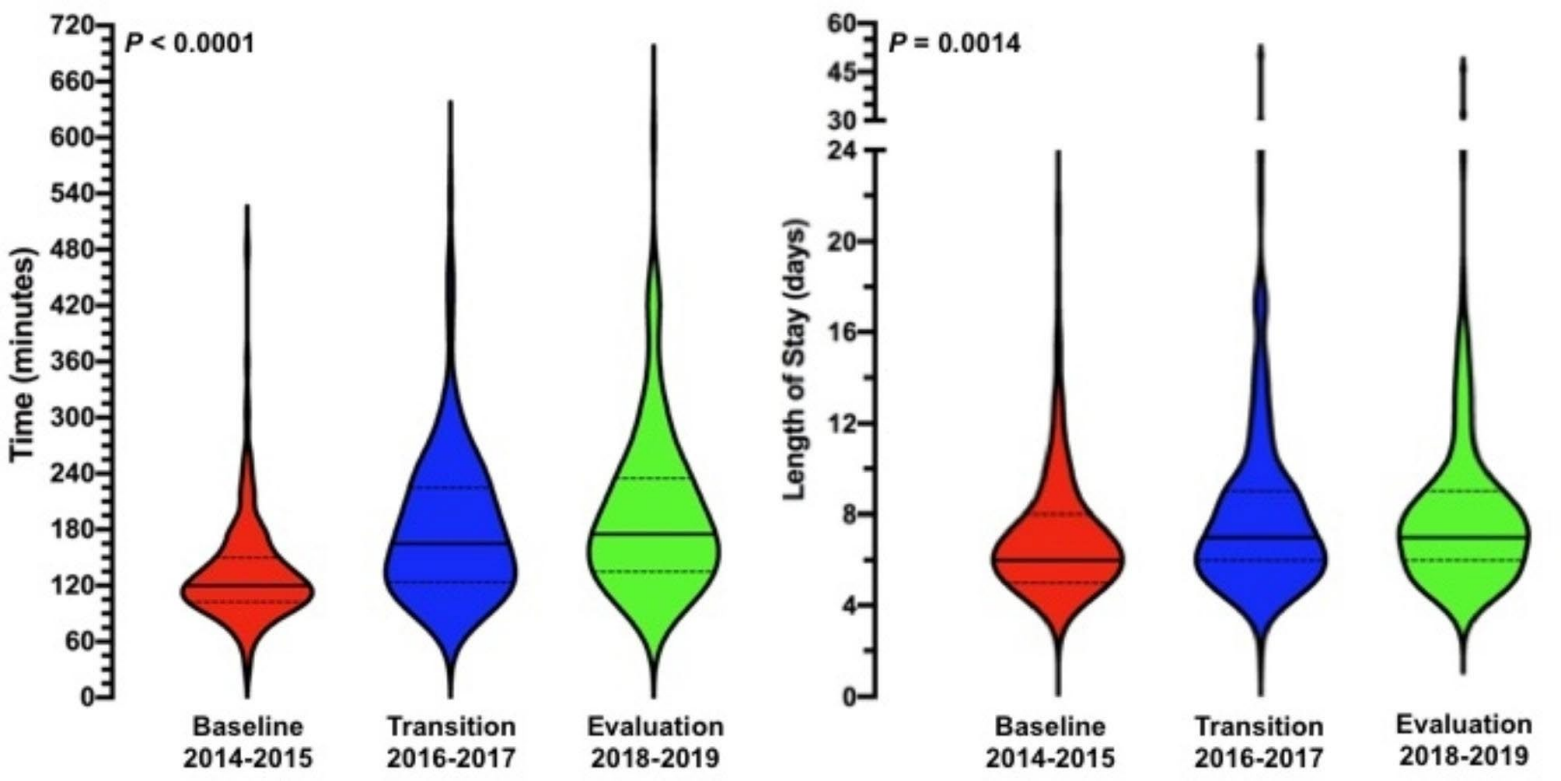
Violin plots of duration of surgery (left panel) and hospital stay (right panel). The abscissa represents time in minutes and days for the left and right panel, respectively. The different time frames are displayed on the ordinate with the red, blue, and green plots representing the number of patients who had cytoreductive surgery during baseline, transition, and evaluation years, respectively

The PDS rate was 26.5, 30.9, and 34.4% for the baseline, transition, and evaluation years, respectively (*P* = 0.254). Whilst there was no difference in the MD, APD, and UAD groups over the years, the median PCI was 4 (1–7), 8 (1–23), and 8 (2–19) when comparing the baseline, transition, and evaluation years, respectively (*P* < 0.0001). Table 2 shows further details of the surgical assessments. The mean operating time during the evaluation years was 197 ± 85 versus 133 ± 55 and 181 ± 75 min during the baseline and transition years, respectively (*P* = 0.0014; Fig. 2).

**Table 2:** Surgical parameters of 559 advanced EOC patients having cytoreductive surgery. A total of 185, 194, and 180 patients were treated during the baseline, transition, and evaluation years, respectively. Numbers are shown either as absolute numbers with percentage unless otherwise indicated

Patients	Baseline Years 2014–2015	Transition Years 2016–2017	Evaluation Years 2018–2019	*P*-value
	n = 185	n = 194	n = 180	
**Surgical cytoreduction**				0.254
PDS	49 (26.5%)	60 (30.9%)	62 (34.4%)	
IDS	136 (73.5%)	134 (69.1%)	118 (65.6%)	
**PCI** ^**a**^ Median (Range)	4 (1–8)	8 (1–24)	8 (2–21)	**< 0.0001**
**Dissemination (GOG)** ^**b**^				**0.408**
MD	17 (9.2%)	17 (8.8%)	19 (10.6%)	
APD	110 (59.2%)	102 (52.6%)	90 (50.0%)	
UAD	58 (31.3%)	75 (38.6%)	71 (39.4%)	
**Surgical Complexity Score (SCS)**				**< 0.00001**
Low (1–3)	135 (73.0%)	110 (56.7%)	74 (41.1%)	
Intermediate (4–7)	49 (26.5%)	68 (35.1%)	84 (46.7%)	
High (8–18)	1 (0.5%)	16 (8.2%)	22 (12.2%)	
**Specialist Surgeons Involved** ^**c**^				**< 0.0001**
One Gyne-Onc	164 (88.6%)	92 (47.4%)	106 (58.9%)	
Two Gyne-Onc’s	9 (4.9%)	72 (37.1%)	40 (22.2%)	
Gyne-Onc & GI	12 (6.5%)	21 (10.8%)	26 (14.5%)	
Gyne-Onc & HPB Gyne-Onc & GI & HPB	0 0	5 (2.6%) 4 (2.1%)	4 (2.2%) 4 (2.2%)	
**Residual Disease (RD)** ^**d**^				**0.0041**
CC 0 (nil RD)	104 (56.2%)	127 (65.5%)	135 (75.0%)	
CC 1 (0 mm < RD < 2.5 mm)	33 (17.8%)	21 (10.8%)	23 (12.8%)	
CC 2 (2.5 mm ≤ RD < 2.5 cm)	32 (17.3%)	29 (14.9%)	17 (9.4%)	
CC 3 (RD ≥ 2.5 cm)	16 (8.7%)	17 (8.8%)	5 (2.8%)	
**Blood Loss (mL)** Mean ± SD	469 ± 321	549 ± 362	551 ± 464	**0.109**

^a^ PCI represents peritoneal cancer index as published previously [23]

^b^ MD (minimal disease), APD (abdominal peritoneal disease), AUD (upper abdominal disease) according to GOG criteria [24]

^c^ Gyne-Onc, GI, and HPB represent Gynecologic Oncologist, Gastro-Intestinal Specialist Surgeon, and Hepato-Billiary Specialist Surgeon, respectively

^d^ CC represents completeness of cytoreduction according to Sugarbaker criteria as published previously [21]

The rate of postoperative HDU/ICU admissions increased from 8.1 to 33.1% (*P* < 0.0001). In addition, unplanned postoperative HDU/ICU admissions decreased from 3.8 to 2.2% for the evaluation years (Table 3). Patients were admitted for 7.0 ± 2.6 days during the baseline years compared to 8.4 ± 4.9 days during the evaluation years (*P* = 0.0014; Fig. 2). No differences in peri-operative morbidity between the baseline, transition, and evaluation years were observed. The intra-operative complications were not recorded. The Clavien-Dindo Grade ≥ IIIA was 9.7, 7.2, and 13.3% for the baseline, transition, and evaluation years, respectively (*P* = 0.142). One-hundred-sixty-seven, 167, and 159 advanced EOC patients were eligible for adjuvant chemotherapy following cytoreduction during the baseline, transition, and evaluation years, respectively. In 10 (6.0%), 13 (7.8%), and 11 (6.9%) patients adjuvant chemotherapy was delayed beyond 6 weeks post-operatively for the respective time intervals (*P* = 0.811). One patient died within 30 days following cytoreductive surgery during the baseline years due to bowel related complications compared to none of the patients during the transition or evaluation years. Three patients died within 60 days after surgery, one in each time interval. None of these cases was directly related to the procedure: instead, these cases were due to complications related to chemotherapy and undisclosed accidents at respectively 45, 48, and 53 days following their procedure. Further postoperative details are displayed in Table 3.

**Table 3:** Postoperative parameters of 559 advanced EOC patients having cytoreductive surgery. A total of 185, 194, and 180 patients were treated during the baseline, transition, and evaluation years, respectively. Numbers are shown either as absolute numbers with percentage unless otherwise indicated

Patients	Baseline Years 2014–2015	Transition Years 2016–2017	Evaluation Years 2018–2019	*P*-value
	n = 185	n = 194	n = 180	
**Postoperative Admissions**				< 0.0001
Regular Ward Admission	170 (91.9%)	138 (71.1%)	124 (68.9%)	
Pre-planned HDU/ITU Admission	1 (0.5%)	19 (9.8%)	32 (17.8%)	
Planned HDU/ITU Admission	7 (3.8%)	33 (17.0%)	20 (11.1%)	
Unplanned HDU/ITU Admission	7 (3.8%)	4 (2.1%)	4 (2.2%)	
**Hospital Stay (days)** Mean ± SD	7.0 ± 2.6	8.3 ± 4.9	8.4 ± 4.9	**0.0014**
**Chemotherapy Regimen** ^**a**^				**0.449**
No Adjuvant CT	18 (9.7%)	27 (13.9%)	21 (11.7%)	
Adjuvant CT	167 (90.3%)	167 (86.1%)	159 (88.3%)	
**Planned Adjuvant CT** ^**b**^				**0.811**
Started ≤ 6w after surgery	157 (94.0%)	154 (92.2%)	148 (93.1%)	
Started > 6w after surgery	10 (6.0%)	13 (7.8%)	11 (6.9%)	
**Peri-operative Morbidity**				**0.142**
Clavien-Dindo Grade 0–2	167 (90.3%)	180 (92.8%)	156 (86.7%)	
Clavien-Dindo Grade ≥ 3 A	18 (9.7%)	14 (7.2%)	24 (13.3%)	
**Postoperative Mortality**				**0.635**
Alive after 60-days	182 (98.4%)	193 (99.5%)	179 (99.4%)	
Death < 30-days	1 (0.5%)	0 (0.0%)	0 (0.0%)	
30-days ≤ Death < 60-days	2 (1.1%)	1 (0.5%)	1 (0.6%)	

^a^ CT represents chemotherapy

^b^ The number of patients who were planned to have adjuvant chemotherapy equaled 167, 167, and 159 during the baseline, transition, and evaluation years, respectively

All ESGO QI’s for advanced stage ovarian cancer surgery were already matched and/or implemented before the baseline years, except QI#1 (complete cytoreduction rate) and QI#10 (complication registration and audit) not fully. With changing our surgical practice, the QI#1 and #10 scores improved. The ESGO QI score [14] improved from 27 for the baseline years to 34 and 34 for the transition and evaluation years respectively (Suppl. Table 1).

**Fig. 3:**
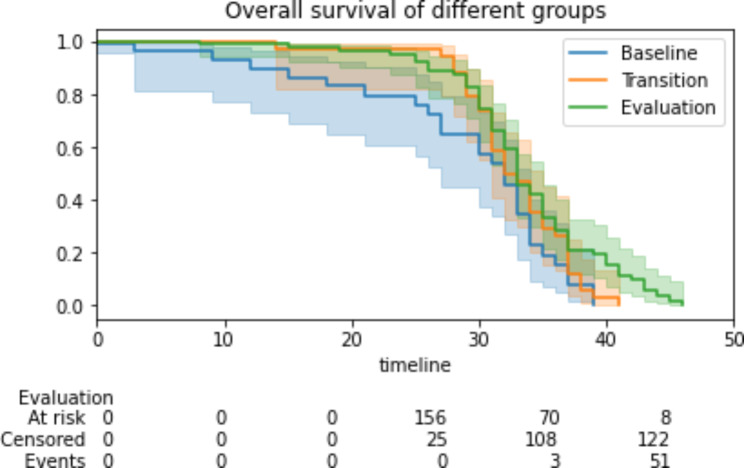
Progression-free-survival analysed by baseline, transition and evaluation groups reflecting different time frames

The median PFS for the entire cohort was 33 months (95% CI 32–34). The median PFS in the baseline, transition, and evaluation groups were 32 months (95% CI 28–36, p: 0.154), 33 months (95% CI 32–35, p: 0.006), and 34 months (95% CI 32–36, p: 0.165), respectively (Fig. 3).

## Discussion

The study reiterates on the benefit of treatment centralization for advanced EOC patients, which most likely relates to the high-quality infrastructure and high levels of expertise. We clearly showed that improving CC 0 rates in advanced stage EOC is achievable without a significant increase in morbidity. Complex multi-visceral surgery is often mandatory for a CC 0 of metastatic tumors. These extended surgical procedures cause longer hospital admissions and often involve HDU/ICU admissions. To manage postoperative morbidity in these patients, their PS should be as optimal as possible.

Our CC 0 rates increased substantially from 57.3% to 74.4%, well above the expected target of 65%. This is comparable to other high volume specialized centers [31]. Although this may be regarded as satisfactory, even higher CC 0 rates may be preferable, yet achievable [32]. Selection bias of patients in our study is considered acceptable, as disease dissemination at the start of the surgical procedure according to the GOG classification was equal in the different time intervals. The higher PCI score during the transition periods was most likely a reflection of more robust assessments of the abdomen and pelvis. Such difference over the years looks surprising but it only reflects collectively the more thorough early intra-operative examination by mobilizing the liver and other organs and opening the pouch of Morrison. We have recently demonstrated that the presence of cancer dissemination in specific anatomical sites, including upper abdomen can be more predictive of CC 0 and survival than the entire PCI [33]. Nevertheless, there was no intention to address individual surgical practice or surgical aggressiveness in this effort.

The increased SCS demonstrates a more aggressive approach in cytoreductive surgery for advanced EOC. However, the SCS does not represent the full domain of surgical cytoreduction for advanced EOC. Mesenteric stripping or resection, pancreas tail resection, cholecystectomy, (partial) gastrectomy, LEER, adrenalectomy, and composite exenteration are examples of procedures we perform to achieve CC 0 resection (albeit not frequently), but do not translate into the SCS. Nevertheless, the SCS reflects the overall complexity of the procedure, the expected postoperative morbidity, and it remains the only externally validated index for surgical complexity in EOC [34]. Notably, the operative time increased by approximately 50%, which agrees with previous findings [11]. The upfront surgical cytoreduction rate increased from 26.5 to 34.4% which unfortunately fell short of our 50% ambition. Our IDS rates of 60-70% were rather high, but not far off the ordinary [35]. It is still debatable how to select for PDS or NACT [36]. Selection criteria to allocate patients to either PDS or NACT have been previously developed [37]. However, their predictive value for routine clinical decision-making is unsatisfactory. Modern data mining technologies, such as Machine Learning appear promising for clinically meaningful improvements of prediction accuracy [38].

A landmark publication by Chi et al. in 2004 showed that improved cytoreduction rates could be established without increasing morbidity rates [11]. However, in that study < 1 cm RD was the aim of the surgical cytoreduction, whereas our aim was to achieve CC 0. The meta-analyses of patients undergoing cytoreductive surgery for advanced stage EOC showed a clear survival benefit for those with CC 0 following cytoreductive surgery [7, 39]. This evidence has shifted the paradigm of surgical management towards CC 0, as the primary outcome measure in cytoreductive surgery of advanced EOC. Yet, to achieve a CC 0 resection rate, more robust preoperative assessments and compliance to prehabilitation are required to prevent an anticipated increased perioperative morbidity and mortality. Facilitating engagement for prehabilitation might require the development of a digital prehabilitation platform to support these women alongside their routine ovarian cancer care.

The observed better preoperative PS of patients during the evaluation years in our study might have been associated with the timely introduced prehabilitation. A recent meta-analysis showed that prehabilitation before major abdominal surgery may improve the preoperative PS and reduce postoperative morbidity [40]. During the transition years in our study, the collaboration with other surgical specialists such as gastro-intestinal (GI) and hepato-biliary (HPB) surgeons was intensified and protocolled. Those Gynecologic Oncologists who have less experience with the required procedures to achieve CC 0 resections were no longer constrained to achieve this in eligible patients. As a result, the utilization of GI and/or HPB surgeons increased from 6.5% to 20%. These observations are coherent with a previous publication on the multidisciplinary surgical approach in advanced ovarian cancer [41]. Advanced stage EOC patients who had unplanned HDU/ICU admissions following their surgical cytoreduction have reportedly poor survival outcomes [42]. Our interdisciplinary meetings to allocate and prioritize HDU/ICU beds prior to the surgical cases may have translated into the observed increased (pre-)planned and decreased unplanned postoperative HDU/ICU admissions of our patients.

Postoperative hospital length of stay increased despite approximately 20% maximizing of our surgical effort. This concurs with a previous study on aggressive surgical cytoreduction [43]. Equally, we failed to confirm the study of Chi et al., that reported no change in hospital stay, albeit their cytoreduction rates improved substantially [11]. One might argue the value of the ERAS program to compensate the anticipated ramifications of a more aggressive surgical approach in terms of postoperative length of stay [44]. In fairness, a difference by one day is of no clinical significance. Also, logistic reasons owing to discharge coordination can negatively impact the outcome of a “medically fit for discharge” patient.

.

Postoperative complications were not substantially increased following a more aggressive surgical approach. This is further strengthened by the similar percentages of patients who had their subsequent adjuvant chemotherapy delayed in the different time intervals of our study. Other groups reported equal morbidity rates in patients after maximal cytoreductive effort compared to those in patients with a more conservative approach [6, 12, 43]. These are important observations, since maximal surgical effort may solely be justified when it does not result in delayed adjuvant chemotherapy treatment and compromised prognosis, especially in the NACT setting [45]. We did not report intra-operative complications because their categorization is not standardized. Alternatively, the internationally agreed way of registering post-operative complications (Clavien-Dindo 3–5 classification) was employed. Our postoperative mortality rate remained unchanged over the years. Although patient characteristics and populations may vary in different studies, the reported mortality rate could be regarded as acceptable [27, 43]. Notably, 9-13% of patients received no adjuvant chemotherapy; this was because of the tumor type. For instance, we do not routinely administer adjuvant chemotherapy in low-grade advanced EOC. In addition, 7% of these patients had a delay of over 6 weeks for starting chemotherapy, not because of surgical morbidity but simply logistics.

Prior to the introduction of the maximal surgical effort concept, our QI score according to the ESGO criteria was 67.5% of the total. Following introduction of maximum surgical effort and morbidity monitored by Clavien-Dindo classification, we achieved our target of > 80% of the total ESGO QI score [14]. This score does not solely measure the surgical outcome but also optimizes the surgical environment, considering the finesses and conditions of decision making in a dynamic environment, as well as enhancing communication and team performance. Solely, with a wide range of supportive measures the preferable standard of surgical care can be achieved [46]. We report a median PFS of 33 months, well above the UK benchmark. We did not observe any significant difference amongst the three groups for PFS, albeit a trend towards improved time-to-relapse was observed. It will be interesting to evaluate the survival outcomes based on the QIs interrogated in this study for the different time intervals. A wider international multi-center study is warranted to correlate these quantifiable measures with patient survivals. Nevertheless, it remains important that, for a safe implementation of complex surgical procedures like maximal surgical effort in EOC, the demands on resources including time, staffing, equipment, continuous professional development, skills, knowledge, space, and funding will be substantiated. The conditions and ramifications of complex surgeries mandate the availability of the aforementioned resources. Therefore, we postulate that complex surgical procedure should solely be carried out in high volume specialized centers, as minimum standards are almost impossible to develop in smaller low volume centers [47].

## Conclusions

A surgical paradigm shift is solely ethically justifiable when it does not translate into higher morbidity. This study focused on the conditions of introducing maximal surgical effort in advanced EOC. Higher CC 0 rates without compromising morbidity was achieved and a trend towards improved PFS was observed. The study also indicated substantial ramifications to this paradigm shift in advanced EOC surgery in terms of the perioperative management and subsequent recovery. A holistic approach supported by Gynecologic Oncologists, other (highly) specialized surgical specialties, specialist nurses, business management of the center, experienced anaesthetic teams, in addition to staff availability was the perquisite for making this surgical transition. The results of this study strengthen our notion that this paradigm shift requires the availability of theatre space, HDU/ICU beds, ward beds, adequate staffing levels, required equipment, finances, and appropriate surgical skills and training. Pivotal is the collaborative approach; assessments of similar approaches in other centers may confirm this concept.

### Electronic supplementary material

Below is the link to the electronic supplementary material.

Supplementary Material 1
