# Supplementary material for: Prerequisites to improve surgical cytoreduction in FIGO stage III/IV epithelial ovarian cancer and subsequent clinical ramifications
Source: J Ovarian Res. 2023 Nov 11;16:214. doi: 10.1186/s13048-023-01303-1 (PMC10638711; doi:10.1186/s13048-023-01303-1)
Supplement: Supplementary file 1 — Supplementary Material 1 [file 13048_2023_1303_MOESM1_ESM.docx]

|  | **Baseline Years** | **Transition Years** | **Evaluation Years** |
| --- | --- | --- | --- |
| **Patients** | **n = 185** | **n = 194** | **n = 180** |
| **QI 1:** |  |  |  |
| Rate complete cytoreductions | 3 | 8 | 8 |
| Rate primary debulking surgery | 0 | 0 | 0 |
| **QI 2:** |  |  |  |
| Surgeries per center | 4 | 4 | 4 |
| Surgeries per surgeon (n > 20) | 5 | 5 | 5 |
| **QI 3:** |  |  |  |
| Surgeries by Gynecologic Oncologist | 3 | 3 | 3 |
| **QI 4:** |  |  |  |
| Participation in clinical trials | 3 | 3 | 3 |
| **QI 5:** |  |  |  |
| Patients discussed in MDT | 2 | 2 | 2 |
| **QI 6:** |  |  |  |
| Preoperative workup | 1 | 1 | 1 |
| **QI 7:** |  |  |  |
| Pre-, intra-, and postoperative management | 1 | 1 | 1 |
| **QI 8:** |  |  |  |
| Elements of operative reports | 3 | 3 | 3 |
| **QI 9:** |  |  |  |
| Elements of pathology reports | 1 | 1 | 1 |
| **QI 10:** |  |  |  |
| Complication registration | 1 | 3 | 3 |
| **Total Score** | **27** | **34** | **34** |

Supplementary Table 1. ESGO QI score [13] for the baseline, transition, and evaluation years. The maximum score equals 40, 80% of the maximum score equals 32. The ESGO QI scores were 27, 34, and 34 for the baseline, transition, and evaluation years, respectively.
